# Astrocytoma-Specific Prognostic Associations of Amyloid-Related Biological Processes

**DOI:** 10.3390/pathophysiology33020030

**Published:** 2026-04-30

**Authors:** Felix Y. Narvaez Irizarry, Tyrel R. Porter, Neisha Ramirez Serrano, Lilia Y. Kucheryavykh

**Affiliations:** Department of Biochemistry, Universidad Central del Caribe, Bayamon, PR 00956, USA; 423fnarvaez@uccaribe.edu (F.Y.N.I.); 122tporter@uccaribe.edu (T.R.P.); 423nramirez@uccaribe.edu (N.R.S.)

**Keywords:** astrocytoma, oligodendroglioma, amyloid precursor protein (APP), amyloid-β clearance

## Abstract

**Background:** Amyloid-related pathways are well studied in neurodegenerative diseases but remain poorly characterized in gliomas. Amyloid-related transcriptional programs in low-grade gliomas (astrocytoma grade II-III) and oligodendrogliomas, and their association with patient survival, were analyzed in this study. **Methods**: Transcriptomic data from 193 grade II-III astrocytomas and 191 oligodendrogliomas were analyzed to evaluate histology-specific expression patterns and prognostic significance. Differential and single-sample gene set enrichment analyses (ssGSEA) were used to calculate per-sample enrichment scores for 30 amyloid-related Gene Ontology biological process gene sets across the combined cohort. These scores were used to compare pathway activity between grade II-III astrocytoma and oligodendroglioma samples. Pathway-level survival analyses were performed for each tumor type using ssGSEA enrichment scores to evaluate associations with overall survival. **Results**: Distinct amyloid-related transcriptional programs were identified between glioma subtypes. Grade II-III astrocytomas showed enrichment of pathways related to amyloid precursor protein (APP) processing and amyloid-β clearance, whereas oligodendrogliomas were enriched in lipid transport and negative regulation of amyloid formation. Survival analyses revealed that higher activity of the positive regulation of APP biosynthetic process and amyloid-β clearance by transcytosis was significantly associated with worse overall survival in grade II-III astrocytoma, but not in oligodendroglioma. Gene-level analyses in astrocytoma demonstrated consistent survival associations across multiple genes within these pathways, supporting coordinated pathway-level effects rather than isolated single-gene prognostic markers. **Conclusions**: Amyloid-related transcriptional programs differ substantially between diffuse glioma subtypes. Increased APP biosynthesis and amyloid-β transcytosis pathways are associated with poorer survival specifically in grade II-III astrocytoma, suggesting a potential role for amyloid metabolism in tumor progression. These findings identify APP-related pathways as candidates for further mechanistic investigation and potential therapeutic targeting in grade II-III astrocytoma.

## 1. Introduction

Diffuse gliomas, including low-grade gliomas (astrocytoma grades II-III according to World Health Organization (WHO) criteria) and oligodendrogliomas, are among the most common primary brain tumors and remain therapeutically challenging because of their infiltrative growth, marked intratumoral heterogeneity, limited drug delivery across the blood–brain barrier, and the establishment of an immunosuppressive tumor microenvironment [1,2,3]. According to current WHO criteria, diffuse gliomas are classified into three molecular types: isocitrate dehydrogenase (IDH)-mutant astrocytomas, IDH-mutant/1p/19q-codeleted oligodendrogliomas, and IDH-wildtype glioblastomas (GBM) [4,5]. These classifications are clinically important, as 1p/19q-codeleted oligodendrogliomas generally demonstrate greater chemosensitivity and more favorable clinical outcomes than IDH-mutant astrocytomas [5,6]. The classical 1p/19q codeletion is a whole-arm deletion involving the short arm of chromosome 1 and the long arm of chromosome 19, most often due to an unbalanced t(1;19)(q10;p10) translocation. This results in hemizygous loss of many genes across both chromosome arms. When present with IDH1/2 mutations, the 1p/19q codeletion defines oligodendroglioma as a distinct molecular class. Almost all such tumors also have IDH mutations and often telomerase reverse transcriptase (TERT) promoter mutations in addition to the codeletion. This underscores the importance of identifying new biomarkers and therapeutic targets better suited to different glioma histologies and physiologies.

Amyloid has traditionally been studied in the context of neurodegenerative diseases. Amyloid beta (Aβ) and its aggregates arise from the proteolysis of amyloid precursor protein (APP) and can dysregulate cellular physiology, leading to neuronal dysfunction or death [7,8,9]. Previous studies have shown that amyloid-β senile plaques are a hallmark of neurodegenerative diseases; however, recent evidence suggests that GBM tumors also exhibit increased amyloid formation and deposition [7,10,11]. This finding raises the possibility that molecular mechanisms involving protein aggregation, typically considered neurodegenerative, may also play a role in tumor biology.

We previously demonstrated direct amyloid accumulation in human GBM specimens using amyloid-binding dyes such as Thioflavin S and Congo red, and further showed by Aβ-specific immunostaining that these deposits contain amyloid-β [11]. Gene expression analyses have shown that elevated APP expression in GBM correlates with distinct tumor phenotypes and with negative enrichment for several immune-related processes, suggesting immune suppression in high-APP tumors [12]. Together, these studies indicate that amyloid-related pathways are active and may influence glioma behavior and the tumor microenvironment [11,12].

Despite these findings, the significance of amyloid-related mechanisms in gliomas remains to be further investigated. To date, no systematic analysis has examined how amyloid metabolism pathways differ across glioma subtypes or how they relate to patient outcomes [12]. To address this gap, this study performed transcriptomic analysis of large cohorts of astrocytoma grade II-III and oligodendroglioma patients, focusing on 30 Gene Ontology biological processes related to amyloid metabolism, APP biosynthesis, Aβ clearance, and amyloid genesis. The goal of the study was to determine whether distinct amyloid-associated transcriptional programs are specific to each oligodendroglioma and astrocytoma grade II-III histology and whether they carry prognostic information. Introducing amyloid biology into glioma research could provide a novel perspective on diffuse glioma pathophysiology and help identify new biomarkers or therapeutic targets.

## 2. Materials and Methods

Transcriptomic data were obtained from the Diffuse Glioma cohort of The Cancer Genome Atlas (TCGA) through the Genomic Data Commons (GDC) via cBioPortal, which included grade 2 and grade 3 glioma tumors [13,14]. Gene expression data consisted of RNA sequencing values normalized to transcripts per million (TPM). Primary diffuse glioma samples were included, comprising 194 grade II-III astrocytomas and 190 oligodendrogliomas. Tumors were classified by histology according to the TCGA grade II-III astrocytoma dataset records.

### 2.1. Differential Expression Analysis

Differential gene expression between grade II-III astrocytoma and oligodendroglioma samples was performed using limma with empirical Bayes moderation, via Expomics [15]. For each gene, log_2_ fold change (log_2_FC), average expression, moderated *t*-statistic, *p*-value, false discovery rate (FDR), and B-statistic were computed. Statistical significance was defined using *p* < 0.05 and FDR < 0.05.

Single-sample gene set enrichment analysis (ssGSEA) was performed on the normalized TPM expression matrix to calculate per-sample enrichment scores for 30 amyloid-related Gene Ontology biological process gene sets across the combined cohort [16]. These enrichment scores were used to assess pathway-level differences between grade II-III astrocytoma and oligodendroglioma samples. Comparisons of ssGSEA scores between histologic subtypes were then performed using a two-sided *t*-test.

### 2.2. Survival Analysis

Survival analyses were conducted separately for grade II-III astrocytoma and oligodendroglioma cohorts. Pathway-level survival analyses were performed using ssGSEA enrichment scores for each amyloid-related gene set. Patients were dichotomized into high- and low-activity groups using the median expression value. Kaplan–Meier survival curves were generated, and group differences were assessed using log-rank tests with FDR adjustment. For pathways significantly associated with survival, gene-level survival analyses were performed for individual genes within the corresponding gene sets using Kaplan–Meier analysis with log-rank testing and multiple-testing correction.

## 3. Results

### 3.1. Distinct Amyloid-Related Transcriptional Programs in Astrocytoma and Oligodendroglioma

**Figure 1:**
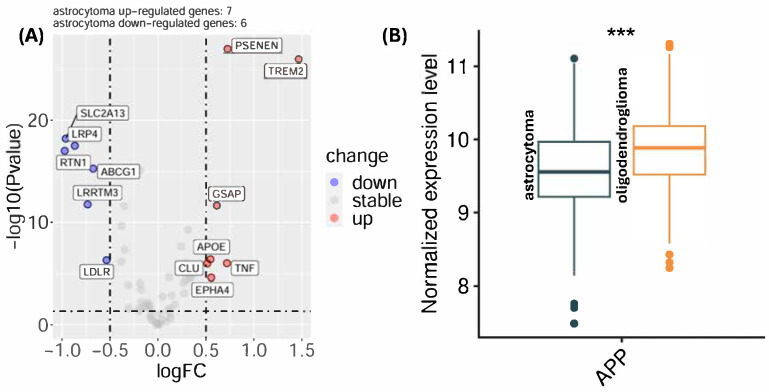
Histology-Specific Differences in Amyloid-β-Related Transcriptional Programs and APP Expression. (**A**) Differential expression analysis of genes belonging to the Gene Ontology biological process amyloid-β metabolic process comparing astrocytoma grade II-III and oligodendroglioma samples. Log_2_ fold change (log_2_FC) values reflect relative expression differences between histologies, with positive values indicating higher expression in astrocytoma grade II-III and negative values indicating higher expression in oligodendroglioma. In the heatmap, red circles indicate genes upregulated in astrocytoma grade II-III compared to oligodendroglioma whereas blue circles are downregulated when compared to oligodendrogliomas. A substantial proportion of genes within this pathway demonstrated statistically significant differential expression (FDR < 0.05), supporting coordinated, histology-specific divergence in amyloid-related transcriptional programs rather than isolated gene-level variation. (**B**) Boxplot showing normalized APP mRNA expression levels in astrocytoma grade II-III and oligodendroglioma samples. Oligodendrogliomas exhibit significantly higher APP expression compared with astrocytoma grade II-III (***, *p* < 0.001). Boxes represent the interquartile range (IQR), center lines indicate the median, whiskers denote the range, and points represent individual samples.

Prior to pathway-level analyses, overall survival was compared between the grade II-III astrocytoma and oligodendroglioma cohorts to establish the prognostic context of the study. Kaplan–Meier analysis demonstrated significantly worse overall survival in grade II-III astrocytoma compared with oligodendroglioma (median survival: 62.1 vs. 95.5 months; log-rank *p* < 0.005; Figure 1). This difference is consistent with the established clinical behavior of these subtypes and confirms that the cohorts used in this study reflect the expected histology-specific survival patterns (Figure 1).

Differential expression analysis using genes from a single gene set, Amyloid Beta Metabolic Process, within Gene Ontology Biological Processes (GOBP), revealed several significantly differentially expressed genes when comparing grade II-III astrocytoma and oligodendroglioma samples (Figure 1, Table 1). Several genes demonstrated statistical significance with log_2_FC > 0.5. Genes with higher expression in grade II-III astrocytoma included TREM2 (log_2_FC = 1.47, FDR = 3.05 × 10^−25^, B = 49.77), PSENEN (log_2_FC = 0.72, FDR = 5.71 × 10^−26^, B = 52.12), TNF (log_2_FC = 0.72, FDR = 3.38 × 10^−6^, B = 4.46), GSAP (log_2_FC = 0.61, FDR = 1.54 × 10^−11^, B = 17.13), EPHA4 (log_2_FC = 0.55, FDR = 1.0 × 10^−4^, B = 1.37), APOE (log_2_FC = 0.55, FDR = 1.71 × 10^−6^, B = 5.32), and CLU (log_2_FC = 0.51, FDR = 3.38 × 10^−6^, B = 4.38). Conversely, genes with higher expression and log_2_FC > 0.5 in oligodendrogliomas included LDLR, ABCG1, LRRTM3, LRP4, SLC2A13, and RTN1. Additional analysis of APP showed a moderate difference as well (log_2_FC = −0.28, moderated *t* = 4.71, FDR < 0.001, B = 3.31).

APP expression differed significantly between grade II-III astrocytoma and oligodendroglioma (Figure 1B). Grade II-III astrocytoma exhibited lower median normalized APP expression compared with oligodendroglioma, and this difference reached strong statistical significance (*p* < 0.001). Although there was some overlap between groups, the interquartile ranges were clearly shifted, indicating a consistent histology-dependent difference in APP transcriptional levels. These findings demonstrate that baseline APP expression varies by glioma subtype, suggesting that amyloid-related transcriptional programs may be differentially regulated across diffuse glioma histologies. The distribution pattern further supports the presence of subtype-specific amyloid-related regulation rather than isolated outlier effects.

To specifically interrogate amyloid-related biology, ssGSEA was performed across all samples using 30 Gene Ontology (GO) biological process gene sets associated with amyloid pathways. Comparison of enrichment scores between grade II-III astrocytoma and oligodendroglioma samples using a two-sided *t*-test revealed significant differences (Table 2). The top pathways enriched in grade II-III astrocytoma were positive regulation of aspartic-type endopeptidase activity involved in amyloid precursor protein catabolic process (*t* = 9.93, *p* < 0.001), regulation of aspartic-type endopeptidase activity involved in amyloid precursor protein catabolic process (*t* = 9.40, *p* < 0.001), and amyloid-beta clearance (*t* = 6.01, *p* < 0.001). In oligodendrogliomas, the highest enrichment was observed for islet amyloid polypeptide processing (*t* = 10.32, *p* < 0.001), negative regulation of amyloid-beta formation (*t* = 2.19, *p* = 0.029), and negative regulation of APP catabolic process (*t* = 2.23, *p* = 0.026).

### 3.2. Prognostic Relevance of Amyloid-Related Biological Processes in Astrocytoma

**Figure 2:**
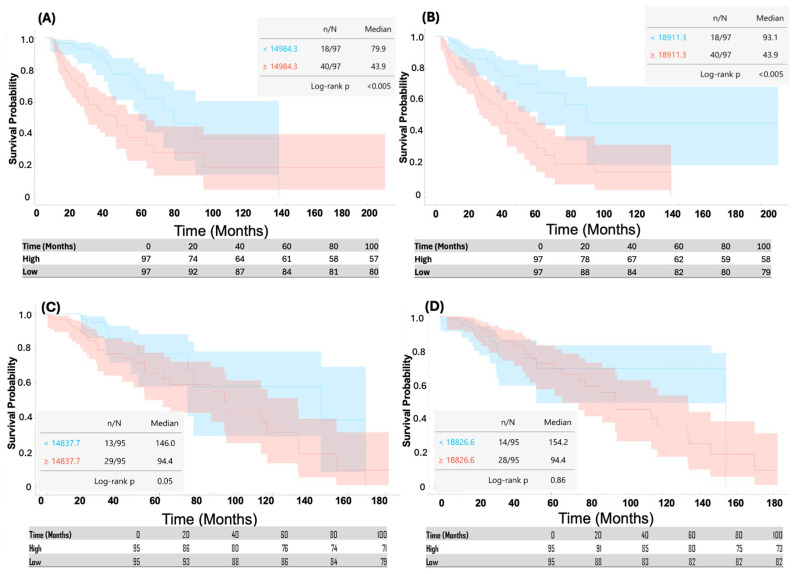
Amyloid-Related Pathway Activity Is Associated with Poor Survival in Astrocytoma Grade II-III. Kaplan–Meier survival curves of astrocytoma grade II-III (**A**,**B**) and oligodendroglioma (**C**,**D**) patients stratified by median ssGSEA enrichment scores for two amyloid-related Gene Ontology biological processes: (**A**,**C**) positive regulation of APP biosynthetic process and (**B**,**D**) amyloid-β clearance by transcytosis. In astrocytoma grade II-III, high pathway activity was significantly associated with reduced overall survival (log-rank *p* = 2.28 × 10^−4^; FDR = 0.004). For positive regulation of APP biosynthesis, median survival was 43.9 months in the high-activity group versus 73.9 months in the low-activity group. For amyloid-β clearance by transcytosis, median survival was 43.9 months in high-activity tumors compared with 93.1 months in low-activity tumors. These findings indicate that increased APP production and enhanced amyloid trafficking programs are associated with more aggressive clinical behavior in astrocytoma grade II-III.

Among astrocytoma grade II-III samples, survival analysis identified two amyloid-related biological processes that were significantly associated with overall survival after FDR correction (Figure 2A,B). The gene set “positive regulation of APP biosynthetic process” was significantly associated with worse survival (log-rank *p* = 2.28 × 10^−4^, FDR = 0.004; χ^2^ = 13.6). Patients with high pathway activity exhibited a median survival of 43.9 months, compared with 73.9 months in the low-activity group. Similarly, amyloid-beta clearance by transcytosis was also significantly associated with survival in grade II-III astrocytoma (log-rank *p* = 2.28 × 10^−4^, FDR = 0.004; χ^2^ = 13.4). Low-activity tumors demonstrated a median survival of 93.1 months, whereas high-activity tumors again showed a median survival of 43.9 months.

In contrast, none of the amyloid-related gene sets demonstrated a significant association with survival in oligodendroglioma (Figure 2C,D). Specifically, for positive regulation of APP biosynthetic process, the log-rank *p*-value was 0.054 (FDR = 0.563; χ^2^ = 3.72). For amyloid-beta clearance by transcytosis, the association was not significant (*p* = 0.811, FDR = 0.955; χ^2^ = 0.03), indicating that the prognostic relevance of amyloid-related biological processes may be histology-specific

To further explore gene-level contributors to the prognostically significant amyloid-related pathways, we performed survival analyses restricted to individual genes belonging to the two amyloid-related gene sets that were significantly associated with overall survival in grade II-III astrocytoma: positive regulation of amyloid precursor protein biosynthetic process and amyloid-beta clearance by transcytosis. Using Kaplan–Meier analysis with log-rank testing and false discovery rate correction, 12 of 21 genes demonstrated significant associations with overall survival (FDR < 0.05). The five genes with the strongest associations were LRPAP1, ITM2C, SOAT1, NECAB1, and NCSTN, further supporting the interpretation that aggregate pathway activity, rather than single-gene expression, underlies the observed prognostic signal in grade II-III astrocytoma (Table 3).

## 4. Discussion

This analysis identified an association between amyloid-related transcriptional programs and overall survival in grade II-III astrocytoma, but not in oligodendroglioma. In grade II-III astrocytoma, elevated activity of the positive regulation of APP biosynthesis and amyloid-β clearance by transcytosis pathways was strongly associated with reduced overall survival. Patients whose tumors exhibited high pathway activity had a median survival of approximately 44 months, compared with 74–93 months in tumors with low enrichment scores. In contrast, neither pathway demonstrated prognostic significance in oligodendroglioma. Notably, analysis of the APP transcript revealed significantly higher expression in oligodendroglioma compared with grade II-III astrocytoma. Despite lower APP mRNA abundance in grade II-III astrocytoma, multiple genes involved in APP processing, amyloid-β clearance, and secretase activity were upregulated in grade II-III astrocytoma relative to oligodendroglioma. These findings indicate that histology-specific differences in amyloid-related biology are not driven solely by APP transcript abundance, but instead reflect coordinated regulation of downstream processing and clearance mechanisms.

Differential expression and pathway enrichment analyses further showed that grade II-III astrocytomas are enriched for APP processing, secretase-related, and amyloid clearance pathways, whereas oligodendrogliomas are enriched for lipid transport genes and negative regulators of amyloid formation. This divergence likely reflects differences in cellular origin and tumor microenvironment between these subtypes. Grade II-III astrocytomas are characterized by stronger inflammatory and microglial signatures [17,18], which may potentiate amyloid metabolism through cytokine-induced APP upregulation and enhanced secretase activity, thereby contributing to a more aggressive phenotype.

The absence of prognostic correlations in oligodendroglioma may indicate fundamental biological differences. Grade II-III astrocytomas (IDH-mutant astrocytic gliomas) and oligodendrogliomas arise from different glial lineages and have distinct genetic and microenvironmental features. Oligodendrogliomas demonstrated enrichment for negative regulation of amyloid-β formation and lipid transport pathways, suggesting a transcriptional program that may limit amyloidogenic stress. Mutant IDH and 1p/19q codeletion contribute to a lineage-specific, less proliferative state and to a relatively less active immune microenvironment compared with grade II-III astrocytoma [19,20,21]. In addition, oligodendroglial lineage cells are intrinsically specialized for lipid metabolism and membrane synthesis [22]. Given that cholesterol composition directly influences β- and γ-secretase activity [23], tightly regulated lipid metabolism in oligodendroglioma may constrain amyloidogenic APP cleavage. Thus, while amyloid-related processes are transcriptionally active in both histologies, only in grade II-III astrocytoma do they appear to integrate with tumor progression programs.

The grade II-III astrocytoma-specific amyloid signature is consistent with literature showing that APP and its proteolytic products influence tumor biology. In breast cancer, APP overexpression promotes proliferation and invasion, whereas APP knockdown reduces tumor growth through modulation of insulin growth factor 1 (IGF-1)/AKT signaling and induction of apoptosis [24,25]. Similarly, glioblastomas with high APP accumulation have been reported to exhibit altered synaptic signaling and immune suppression programs [12]. These findings suggest that APP may actively modulate tumor behavior. Mechanistically, cleavage of APP by β- and γ-secretases produces amyloid-β peptides as well as the APP intracellular domain (AICD), which have been shown to regulate transcription and activate pro-survival pathways [26,27,28]. APP fragments have also been shown to activate phosphatidylinositol 3-kinase (PI3K)/AKT signaling and promote survival and invasion [26]. Therefore, increased positive regulation of APP biosynthesis in grade II-III astrocytoma may enhance production of oncogenic APP fragments, contributing to tumor progression.

Increased expression of genes involved in amyloid-β clearance by transcytosis was also associated with poor survival in grade II-III astrocytoma. Although clearance might be expected to be protective, elevated expression of these genes may reflect increased amyloid burden and compensatory trafficking responses. Receptor-mediated transcytosis, largely mediated by low-density lipoprotein receptor-related protein 1 (LRP1) family receptors across the blood–brain barrier, plays a key role in amyloid transport [29,30,31]. Dysregulation of these pathways could alter extracellular amyloid distribution within the tumor microenvironment.

The role of Aβ in gliomas appears to depend on its aggregation state and microenvironmental context. Previous reports indicate that oligomeric Aβ activates microglia through innate immune receptors and promotes a pro-phagocytic phenotype, whereas modulation of APP processing through Beta-site Amyloid Precursor Protein Cleaving Enzyme 1 (BACE1) inhibition can reprogram tumor-associated macrophages toward a more phagocytic, anti-tumor state [32,33]. Together, these findings indicate that amyloid metabolism can either restrain or promote tumor growth depending on immune context and processing dynamics. Our data suggest that in grade II-III astrocytoma, sustained upregulation of APP biosynthesis and trafficking correlates with worse outcomes, potentially reflecting chronic activation of oncogenic APP signaling or maladaptive immune remodeling rather than effective tumor suppression.

Among the gene-level associations, LDL receptor-related protein-associated protein 1 (LRPAP1), sterol O-acyltransferase 1 (SOAT1), and nicastrin (NCSTN) emerged as notable adverse markers. LRPAP1 encodes receptor-associated protein (RAP), an inhibitor of LDL receptor family members, and extracellular LRPAP1 has been shown to inhibit microglial phagocytosis and amyloid uptake [29,34]. SOAT1 regulates cholesterol esterification, and because membrane cholesterol modulates β- and γ-secretase activity, increased SOAT1 expression may favor amyloidogenic APP cleavage [23,35,36,37]. NCSTN is an essential component of the γ-secretase complex, and increased NCSTN expression may enhance cleavage of substrates such as APP and NOTCH receptors, promoting amyloid-β production and oncogenic signaling [38]. Integral membrane protein 2C (ITM2C), which binds APP and inhibits amyloid-β production in neuronal systems [39,40], also predicted worse survival in grade II-III astrocytoma, possibly reflecting tumor-specific functions or compensatory upregulation in aggressive tumors.

Collectively, these findings suggest that grade II-III astrocytomas with heightened APP production, enhanced secretase activity, altered cholesterol metabolism, and modified amyloid trafficking exhibit transcriptional programs associated with aggressive clinical behavior, whereas oligodendrogliomas appear transcriptionally buffered against amyloidogenic stress.

The study has several limitations. First, the analyzed TCGA grade II-III astrocytoma cohort included both grade 2 and grade 3 astrocytomas, and the oligodendroglioma cohort also included grade 2 and grade 3 tumors, but survival analyses were performed by histologic subtype without additional stratification by grade. In addition, these data were derived from a cohort annotated before the updated WHO 2021 classification guidelines, which may limit direct alignment with current molecular diagnostic criteria. Because tumor grade and updated classification are important determinants of prognosis and disease definition, future studies should determine whether these amyloid-related associations remain significant after grade-adjusted analysis using cohorts classified according to current WHO criteria.

## 5. Conclusions

This study demonstrates that amyloid-related transcriptional programs are differentially regulated across diffuse glioma subtypes and identifies a strong grade II-III astrocytoma-specific association between increased APP biosynthesis and amyloid-β transcytosis pathways and poor overall survival. The coordinated prognostic effect across multiple pathway components supports biological relevance rather than isolated gene associations. The absence of similar associations in oligodendroglioma underscores fundamental subtype-specific differences in amyloid engagement. Together, these findings position amyloid metabolism as a potentially important contributor to grade II-III astrocytoma aggressiveness and highlight APP-related pathways as candidates for further mechanistic investigation and therapeutic exploration.

## Figures and Tables

**Table 1:** Differentially expressed genes comparing astrocytoma grade II-III with oligodendroglioma samples with log_2_FC.

Gene Name	Log_2_FC	AveExpr	Moderated t-Statistic	*p*-Value	Adj. *p*-Value	B-Statistic
TREM2	1.4663	6.7361	−11.5543	1.03 × 10^−26^	3.05 × 10^−25^	49.7743
PSENEN	0.7248	4.6984	−11.8271	9.67 × 10^−28^	5.71 × 10^−26^	52.1229
TNF	0.7184	2.1606	−4.9613	1.05 × 10^−6^	3.38 × 10^−6^	4.462
GSAP	0.6128	2.0721	−7.2468	2.35 × 10^−12^	1.54 × 10^−11^	17.1335
EPHA4	0.5548	3.937	−4.2525	0	0.0001	1.3709
APOE	0.5471	11.725	−5.1419	4.34 × 10^−7^	1.71 × 10^−6^	5.3166
CLU	0.5135	11.7292	−4.944	1.15 × 10^−6^	3.38 × 10^−6^	4.3813
EFNA1	0.3486	5.1616	−4.3098	0	0.0001	1.605
BACE2	0.3329	2.2465	−4.2243	0	0.0001	1.2568
PICALM	0.3122	6.5727	−6.3644	5.58 × 10^−10^	2.74 × 10^−9^	11.7816
ABCA7	0.291	2.0984	−4.1639	0	0.0001	1.0148
IFNGR1	0.281	6.6879	−4.6169	5.32 × 10^−6^	0	2.9067
TMED10	0.2777	7.5521	−4.9505	1.11 × 10^−6^	3.38 × 10^−6^	4.4117
SORL1	0.2718	5.9359	−3.3546	0.0009	0.0017	−1.9164
ROCK1	0.2666	4.1065	−3.9693	0.0001	0.0002	0.2567
GSK3A	0.2409	4.6827	−5.702	2.36 × 10^−8^	1.07 × 10^−7^	8.1352
BIN1	0.1756	6.5429	−2.5515	0.0111	0.0182	−4.2327
APH1A	0.1432	7.277	−3.217	0.0014	0.0026	−2.3558
NTRK2	0.1305	8.0436	−1.1634	0.2454	0.2896	−6.7833
SP1	0.124	4.8932	−2.1976	0.0286	0.0392	−5.0604
CASP3	0.1232	5.12	−1.7518	0.0806	0.1034	−5.9311
IGF1	0.1172	0.3461	−4.2907	0	0.0001	1.5266
NCSTN	0.105	6.6132	−2.0578	0.0403	0.054	−5.3541
SPON1	0.0861	6.6552	−0.6905	0.4903	0.5366	−7.2203
IFNG	0.0472	0.0728	−3.1598	0.0017	0.003	−2.5335
RELA	0.042	5.9621	−0.9119	0.3624	0.4192	−7.0433
LRP1	0.0214	7.3155	−0.3011	0.7635	0.8044	−7.4131
PSEN2	0.018	0.19	−1.7088	0.0883	0.1108	−6.0049
NAT8B	0.0047	0.4082	−0.1362	0.8918	0.9231	−7.4491
IDE	0.002	2.485	−0.0478	0.9619	0.9785	−7.4572
APOA1	−0.0009	0.6415	0.0174	0.9861	0.9861	−7.4582
PSEN1	−0.0196	5.063	0.3817	0.7029	0.754	−7.3856
GGA3	−0.0303	4.8374	0.6892	0.4911	0.5366	−7.2211
CD36	−0.0657	0.7431	0.89	0.374	0.4243	−7.0629
BECN1	−0.0744	4.5119	1.6509	0.0996	0.1224	−6.1013
ROCK2	−0.0818	4.4753	1.3736	0.1704	0.2051	−6.5179
APEH	−0.0848	6.0395	2.252	0.0249	0.0365	−4.941
REN	−0.0892	0.2541	2.5214	0.0121	0.0193	−4.3077
DYRK1A	−0.108	3.4913	2.7017	0.0072	0.0121	−3.8455
APH1B	−0.1158	4.823	2.3719	0.0182	0.0275	−4.6678
BACE1	−0.1446	5.0404	2.4313	0.0155	0.0241	−4.5271
CSNK1E	−0.1675	6.0528	2.7406	0.0064	0.0111	−3.7417
RTN4	−0.174	8.0353	3.5951	0.0004	0.0007	−1.1066
PIN1	−0.187	5.691	3.9388	0.0001	0.0002	0.1409
CHRNA7	−0.1877	0.3143	8.4024	8.56 × 10^−16^	7.22 × 10^−15^	24.9201
ACE	−0.1948	1.7137	1.9913	0.0471	0.0618	−5.4871
ABCA2	−0.1989	6.6356	2.2161	0.0273	0.0383	−5.02
HAP1	−0.3027	3.9985	2.2451	0.0253	0.0365	−4.9563
MGAT3	−0.3084	5.1243	3.8491	0.0001	0.0003	−0.1948
PRNP	−0.3489	8.7122	5.5621	5.00 × 10^−8^	2.11 × 10^−7^	7.4079
RTN3	−0.3511	8.7878	7.2244	2.72 × 10^−12^	1.61 × 10^−11^	16.991
MME	−0.3834	0.5973	6.5135	2.30 × 10^−10^	1.23 × 10^−9^	12.6473
RTN2	−0.3836	3.8613	5.0391	7.21 × 10^−7^	2.5 × 10^−6^	4.827
LDLR	−0.5357	3.5899	5.1036	5.25 × 10^−7^	1.94 × 10^−6^	5.1333
ABCG1	−0.6757	4.7207	8.4604	5.63 × 10^−16^	5.54 × 10^−15^	25.3325
LRRTM3	−0.7331	4.3203	7.2876	1.80 × 10^−12^	1.33 × 10^−11^	17.3944
LRP4	−0.8667	6.6298	9.1563	3.22 × 10^−18^	4.76 × 10^−17^	30.4255
SLC2A13	−0.9613	3.6932	9.367	6.42 × 10^−19^	1.26 × 10^−17^	32.019
RTN1	−0.972	7.4298	9.0057	1.01 × 10^−17^	1.19 × 10^−16^	29.3012

**Table 2:** Differential Enrichment of Amyloid-Related Gene Sets Between Astrocytoma Grade II-III and Oligodendroglioma. Single-sample gene set enrichment analysis (ssGSEA) comparing 30 amyloid-related Gene Ontology biological processes between astrocytoma grade II-III and oligodendroglioma. astrocytoma grade II-III demonstrated significant enrichment of gene sets related to APP catabolic regulation, amyloid-β clearance, and positive regulation of APP biosynthesis, whereas oligodendrogliomas were enriched for islet amyloid polypeptide processing and negative regulation of amyloid formation (two-sided *t*-test). Reported *t*-values and *p*-values reflect histology-specific differences in pathway activity, highlighting distinct amyloid-associated transcriptional programs across diffuse glioma subtypes.

Gene Set Name	Higher in	*t* Value	*p* Value
Islet Amyloid Polypeptide Processing	Oligodendroglioma	10.316	0
Positive Regulation of Aspartic-type Endopeptidase Activity Involved in Amyloid Precursor Protein Catabolic Process	Astrocytoma	9.932	0
Regulation of Aspartic-type Endopeptidase Activity Involved in Amyloid Precursor Protein Catabolic Process	Astrocytoma	9.403	0
Amyloid-beta Clearance	Astrocytoma	6.013	0
Amyloid-beta Clearance by Transcytosis	Astrocytoma	5.302	0
Positive Regulation of Amyloid Precursor Protein Catabolic Process	Astrocytoma	4.346	0
Positive Regulation of Amyloid Precursor Protein Biosynthetic Process	Astrocytoma	4.33	0
Positive Regulation of Amyloid-beta Clearance	Astrocytoma	4.279	0
Regulation of Amyloid-beta Clearance	Astrocytoma	4.182	0
Cellular Response to Amyloid-beta	Astrocytoma	3.339	0.001
Amyloid-beta Clearance by Cellular Catabolic Process	Oligodendroglioma	2.911	0.004
Negative Regulation of Amyloid Precursor Protein Catabolic Process	Oligodendroglioma	2.231	0.026
Negative Regulation of Amyloid-beta Formation	Oligodendroglioma	2.185	0.029
Negative Regulation of Amyloid-beta Clearance	Astrocytoma	2.177	0.03

**Table 3:** Individual genes involved in the regulation of amyloid precursor protein biosynthesis and amyloid-β clearance by transcytosis including those significantly associated with overall survival in astrocytoma grade II-III (Kaplan–Meier analysis divided by median values with log-rank test and FDR correction).

Genes	*p*-Value	FDR	HR	95%CI
LRPAP1	≤0.001	≤0.001	3.98	2.25–7.03
ITM2C	≤0.001	≤0.001	3.19	1.8–5.63
SOAT1	≤0.001	0.002	2.78	1.54–5.01
NECAB1	≤0.001	0.002	2.61	1.49–4.59
NCSTN	≤0.001	0.002	2.62	1.49–4.63
PAWR	≤0.001	0.002	2.51	1.45–4.34
BACE2	≤0.001	0.003	2.43	1.4–4.21
AGO2	≤0.001	0.003	2.52	1.41–4.48
PICALM	0.002	0.006	2.37	1.33–4.22
CLTC	0.003	0.006	2.27	1.31–3.94
LRP1	0.006	0.12	2.1	1.22–3.61
RAB5A	0.012	0.021	2.0	1.15–3.47
NECAB3	0.038	0.062	1.73	1.02–2.91
ABCA2	0.05	0.076	1.68	0.99–2.85
RAB11A	0.073	0.102	1.63	0.95–2.8
ABCA7	0.207	0.272	1.4	0.83–2.36
AATF	0.249	0.307	0.74	0.44–1.24
RAB11B	0.306	0.356	1.31	0.78–2.21
ITM2A	0.458	0.506	1.22	0.72–2.05
NECAB2	0.689	0.724	0.9	0.54–1.51
ITM2B	0.958	0.958	0.99	0.59–1.65

## Data Availability

The data generated in this study are available within the article.

## Abbreviations

The following abbreviations are used in this manuscript:

Aβ	Amyloid beta
AICD	APP intracellular domain
APH-1	Anterior Pharynx Defective 1
APP	Amyloid Precursor Protein
BACE1	Beta-site Amyloid Precursor Protein Cleaving Enzyme 1
CIC	capicua transcriptional repressor
FDR	False Discovery Rate
FUBP1	far upstream element-binding protein 1
GBM	Glioblastoma
GDC	Genomic Data Commons
GO	Gene Ontology
GOBR	Gene Ontology Biological Process
IDH	Isocitrate Dehydrogenase
IGF-1	Insulin Growth Factor 1
IQR	Interquartile Range
ITM2C	Integral Membrane Protein 2C
log_2_FC	log_2_ fold change
LRP1	low-density lipoproteins
LDL	low-density lipoprotein receptor-related protein 1
LRPAP1	LDL Receptor-Related Protein-Associated Protein 1
NCSTN	Nicastrin
PEN-2	Presenilin Enhancer 2
PI3K	phosphatidylinositol 3-kinase
PSEN1/2	presenilin
RAP	Receptor-Associated Protein
ssGSEA	Single-sample gene set enrichment analysis
SOAT1	Sterol O-Acyltransferase 1
TCGA	The Cancer Genome Atlas
TERT	Telomerase Reverse Transcriptase
TLR	Toll-like receptors
TPM	Transcripts per million
